# Developmental Changes in HCN Channel Modulation of Neocortical Layer 1 Interneurons

**DOI:** 10.3389/fncel.2018.00020

**Published:** 2018-01-30

**Authors:** Andrew S. Bohannon, John J. Hablitz

**Affiliations:** Department of Neurobiology, University of Alabama at Birmingham, Birmingham, AL, United States

**Keywords:** HCN channels, interneuron development, layer 1, medial agranular cortex, synaptic integration

## Abstract

Layer 1 (L1) interneurons (INs) play a key role in modulating the integration of inputs to pyramidal neurons (PNs) and controlling cortical network activity. Hyperpolarization-activated, cyclic nucleotide-gated, non-specific cation (HCN) channels are known to alter the intrinsic and synaptic excitability of principal components (PCs) as well as select populations of GABAergic INs. However, the developmental profile and functional role of HCN channels in diverse L1 IN populations is not completely understood. In the present study, we used electrophysiological characterization, in conjunction with unbiased hierarchical cluster analysis, to examine developmental modulation of L1 INs by HCN channels in the rat medial agranular cortex (AGm). We identified three physiologically discrete IN populations which were classified as regular spiking (RS), burst accommodating (BA) and non-accommodating (NA). A distinct developmental pattern of excitability modulation by HCN channels was observed for each group. RS and NA cells displayed distinct morphologies with modulation of EPSPs increasing in RS cells and decreasing in NA cells across development. The results indicate a possible role of HCN channels in the formation and maintenance of cortical circuits through alteration of the excitability of distinct AGm L1 INs.

## Introduction

In mature neocortex, a small population of GABAergic cells comprises the entirety of layer 1 (L1) neurons (DeFelipe and Jones, 1988; Winer and Larue, 1989; Hestrin and Armstrong, 1996; Zhou and Hablitz, 1996b). Cajal-Retzius cells (CR) in L1 are one of the earliest identifiable cell-types in the neocortex (Bradford et al., 1978; Chun and Shatz, 1989) and are critical for proper cortical development, specifically cortical layering (Peters and Jones, 1984; Ogawa et al., 1995). CR cells exhibit a unique morphology, featuring a prominent tapering dendrite and ovoid soma, and can be identified by their expression of reelin. However, CR cells are no longer present by the end of the second post-natal week (del Río et al., 1995; Zhou and Hablitz, 1996b; Soda et al., 2003; Chowdhury et al., 2010). Although the exact mechanism behind CR cell elimination is unclear, it has been suggested that they are removed from the cortex via apoptosis (Kirischuk et al., 2014), leaving a sparse diverse population of interneurons (INs) which remains in L1 throughout maturity. We have previously shown that L1 INs display robust “sag” responses upon membrane hyperpolarization, indicative of the presence of an inwardly-rectifying hyperpolarization-activated current (*I*_h_; Zhou and Hablitz, 1996b; Wu and Hablitz, 2005). However, there has been no characterization of developmental changes in these responses or the functional impact of *I*_h_ on the excitability of those cells. Given L1’s critical role in establishing cortical lamination and circuitry, the functional impact of hyperpolarization-activated, cyclic nucleotide-gated, non-specific cation (HCN) channels on L1 INs during development is potentially important. Despite the absence of labeling with cell markers typically used to classify GABAergic neurons in other cortical layers (Kawaguchi and Kubota, 1997; Xu et al., 2010; Pfeffer et al., 2013), L1 INs have been shown to consist of distinct subclasses based on anatomical and electrophysiological parameters (Jiang et al., 2013; Ma et al., 2014; Lee et al., 2015). Several studies have divided mature L1 INs into late-spiking and non-late-spiking groups for characterization (Chu et al., 2003; Cruikshank et al., 2012). However, recent studies have demonstrated morphological and physiological diversity of L1 INs which is not accurately reflected when using late-spiking as the sole distinguishing feature (Kubota et al., 2011; Jiang et al., 2013). In order to better understand the diversity of L1 IN subtypes, we have examined specific mechanisms regulating the excitability and activity of L1 INs throughout development.

Changes in the excitability of discrete cell populations can disrupt cortical network dynamics and subsequently alter normal cortical function (Cossart et al., 2001; Cobos et al., 2005; Trevelyan et al., 2006; Yizhar et al., 2011). Alterations in several voltage-dependent currents have been shown to influence the excitability of neurons. In particular, studies have characterized the role of HCN channels in influencing the excitability of cortical cell populations (Robinson and Siegelbaum, 2003; Biel et al., 2009; Albertson et al., 2011, 2013). Further implicating HCN channels in regulatory control of network activity, knockout of HCN1 (Huang et al., 2009; Santoro et al., 2010) and HCN2 (Ludwig et al., 2003) has been observed to produce a decreased seizure threshold and absence seizures, respectively. Multiple experimental epilepsy models and human epilepsy cases have reported alterations in the presence and/or function of HCN channels (Dyhrfjeld-Johnsen et al., 2009; Baruscotti et al., 2010; Reid et al., 2012; Shah et al., 2012). The cell type-specific basis for these observed changes in HCN channel expression/function has yet to be determined.

The medial agranular cortex (AGm) is a strip of cortex lying between the anterior cingulate and lateral agranular cortex (AGl). The AGm is most strongly differentiated from surrounding cortical areas by its cortical connectivity patterns, with AGm sending projections to primary motor cortex and sensory cortical areas (Reep et al., 1987, 1990; Gu et al., 1999; Jeong et al., 2016). Based on these connectivity patterns, the AGm is considered a homolog of the premotor and supplementary motor areas of the primate cortex (Passingham et al., 1988; Sul et al., 2011). Accordingly, activation of AGm efferent fibers incites little to no motor activity (Donoghue and Wise, 1982; Sanderson et al., 1984; Neafsey et al., 1986). Rather, AGm has been demonstrated to be critical for the selection of informed or value-based actions (Kargo et al., 2007; Ostlund et al., 2009; Erlich et al., 2011; Murakami et al., 2014; Li et al., 2015). Given that L1 INs regulate information processing in cortical circuits (Cauller, 1995; Shlosberg et al., 2006; Palmer et al., 2012a; Jiang et al., 2013), regulation of the excitability of L1 cells in AGm may be crucial for maintenance of cortical output.

Identification of mechanisms which regulate the intrinsic and synaptic excitability of L1 neurons is pivotal to understand the network dynamics underlying information processing. With multiple brain regions densely innervating principal component (PC) dendrites in L1 (Cauller, 1995; Cauller et al., 1998; Rubio-Garrido et al., 2009), GABAergic cells in this layer are uniquely positioned to integrate inputs and finely control cortical output (Petreanu et al., 2009). Intrinsic neuronal excitability (Magee, 2000) and the time course of distally evoked EPSPs are increased following HCN channel inhibition. Temporal summations of EPSPs is also enhanced (Williams and Stuart, 2000; Berger et al., 2001) which can allow somatic membrane potentials to move significantly closer to threshold (Berger et al., 2001). Identifying the role of HCN channels in modulating excitability and synaptic integration in L1 INs will increase our understanding of the basic mechanisms governing cortical network activity which underlies information processing. To this end, we used whole-cell electrophysiological recordings to determine the extent to which HCN channels affect the intrinsic and synaptic excitability of AGm L1 INs throughout development. We have identified physiologically discrete subsets of L1 INs with distinct developmental patterns of modulation by HCN channels.

## Materials and Methods

### Ethics Statement

All experiments were performed in accordance with the National Institutes of Health Guide for the Care and Use of Laboratory Animals. Research protocols were approved by the University of Alabama at Birmingham Institutional Care and Use Committee. All available measures were taken to minimize pain or discomfort for research subjects.

### Slice Preparation

Acute cortical slices containing the AGm were prepared from vesicular GABA transporter (VGAT)-Venus-A-expressing Wistar rats (Uematsu et al., 2008). Animals were anesthetized with isoflurane and decapitated. The brain was quickly removed and immediately placed in ice-cold oxygenated (95% O_2_/5% CO_2_, pH 7.4) cutting solution consisting of (in mM): 135 N-Methyl-D-glucamine, 23 NaHCO_3_, 1.5 KH_2_PO_4_, 0.4 ascorbic acid, 1.5 KCl, 0.5 CaCl_2_, 3.5 MgCl_2_ and 10 D-glucose (Tanaka et al., 2008). Coronal brain slices (300 μm thick) were made using a Microm HM 650 vibratome (Microm; Walldorf, Germany). Slices were stored in saline containing (in mM) 125 NaCl, 26 NaHCO_3_, 1.25 NaH_2_PO_4_, 3.5 KCl, 2.0 CaCl_2_, 2.0 MgCl_2_ and 10 D-glucose at 37°C for 45 min, then kept at room temperature for a minimum of 1 h until recording.

### Whole Cell Recording

Individual slices were transferred to a submerged recording chamber mounted on the stage of a Zeiss Axio Examiner D1 (Carl Zeiss Inc., Thornwood, NY, USA) microscope, equipped with Dodt contrast optics, a 40×-water immersion lens and infrared illumination to view neurons in the slices. The recording chamber was continuously perfused with oxygenated saline (3 ml/min at 30°C). Borosilicate patch electrodes had an open tip resistance of 3–6 MΩ when filled with an intracellular solution containing (in mM): 125 K-gluconate, 10 KCl, 10 HEPES, 10 creatine-PO_4_, 2 Mg-ATP, 0.2 Na-GTP, 0.5 EGTA, which had an adjusted pH and osmolarity of 7.3 and 290, respectively. For a subset of control experiments, a cesium-based internal solution was used containing (in mM): 129 CsCl, 10 HEPES, 2 Mg-ATP, 0.2 Na-GTP, 10 EGTA, which had an adjusted pH and osmolarity of 7.3 and 290, respectively. Tight seals of 1 GΩ or greater were obtained under visual guidance before breaking into whole-cell mode. INs in L1 were identified by their proximity to the pial surface and the low density of cells within L1 compared to layer 2/3.

### Intrinsic Properties

Several intrinsic properties of neurons, specifically: resting membrane potential (RMP), action potential (AP) height, AP half-width, AP threshold, after-hyperpolarization (AHP) amplitude, time to AHP peak (tAHP), initial firing frequency, and frequency adaptation were recorded for use in cluster analysis. Input resistance (*R*_IN_) was not included as a variable for cluster analysis as the drastic developmental changes in *R*_IN_ caused cells to be grouped solely based on the postnatal day at which they were recorded. RMP was calculated from a minimum of five records at the membrane potential in the absence of network activity or stimulation. AP and AHP properties were calculated from the first AP fired in response to depolarizing current injected in 50 pA steps. AP threshold was identified as the potential at which rapid membrane depolarization began. AP height was calculated from threshold to the peak amplitude before repolarization, and AP half-width was defined as the width of the AP at half-maximum amplitude. AHP amplitude and tAHP were defined as the most hyperpolarized potential reached following an AP and the time to peak following return to RMP upon repolarization, respectively. Initial firing frequency and frequency adaptation were calculated from responses to current injection double the rheobase amplitude. Initial firing frequency was calculated as the reciprocal of the first interspike interval (ISI), and frequency adaptation was defined as the percent change in ISI from the first to last spikes (1st ISI–last ISI/1st ISI). *R*_IN_ was calculated using Ohm’s law from the voltage deflection in response to a −50 pA current injection.

### Morphological Reconstruction

Biocytin (0.5%; Sigma, St. Louis, MO, USA) or Alexa Fluor 594 hydrazide (0.075%; Molecular Probes, Eugene, OR, USA) was added to the intracellular solution in some experiments for *post hoc* morphological analysis. Slices with biocytin-filled cells were processed as previously described (Zhou and Hablitz, 1996a). Slices with Alexa Fluor filled cells were fixed in paraformaldehyde at 4°C for 48 h then mounted to slides for imaging. Fluorescently labeled cells were imaged using a Zeiss LSM 510 confocal microscope (Carl Zeiss Inc., Thornwood, NY, USA) using a 605/670 bandpass emission filter. Images were acquired using Zen software (Zen Software Inc., Trumbull, CT, USA) and further processed using ImageJ (U.S. NIH, Bethesda, MD, USA) and Photoshop (Adobe Systems Inc., San Jose, CA, USA). L1 INs with an axon extending >200 μm from the pial surface (or >100 μm into layer II/III) were classified as deep-projecting whereas cells with axons projecting laterally within L1 were termed horizontally projecting. Using that criteria, a chi-squared test was performed to determine if neuronal physiology and type of axon projection are independent properties.

### Data Acquisition and Analysis

Whole-cell recordings were obtained using an ELC-03XS npi bridge balance amplifier (npi Electronic GmbH, Tamm, Germany). Signals were acquired using Clampex software with a Digidata 1322A interface (Molecular Devices). Evoked responses were digitized at 10 kHz, filtered at 2 kHz and analyzed using Clampfit 9.0 software (Molecular Devices). Synaptic responses were evoked using a nichrome bipolar electrode positioned in L2, ~100 μm from the recording electrode, using 10–100 μA current pulses of 100 μs duration. EPSP summation was calculated as the percent change in the amplitude of the fifth evoked event relative to the amplitude of the first event. Area under the curve (AUC) of evoked trains was calculated from the onset of the first stimulation until return to RMP following the fifth stimulation. AUC was normalized to the amplitude of the first EPSP to account for changes in input, as stimulus intensity was kept constant for pre- and post-drug trials. Both summation and AUC were initially analyzed across all frequencies, using a post-test to identify frequency-specific effects. In a set of control experiments, EPSCs were recorded from L1 INs held at −70 mV to eliminate voltage-dependent changes in HCN channel activity. Miniature event analysis was performed using MiniAnalysis (Synaptosoft). An equal number of consecutive events was taken from each recorded cell for analysis.

### Drugs and Drug Application

Bicuculline-methiodide (10 μM; Abcam, Cambridge, MA, USA) or SR95531-hydrobromide (10 μM; Tocris, Ellisville, MO, USA) was present in the saline for all experiments to block GABA_A_ receptor mediated synaptic transmission. After recording control responses, 4-Ethylphenylamino-1,2-dimethyl-6-methylaminopyrimidinium chloride (20 μM; ZD7288; Tocris Bioscience, Ellisville, MO, USA) was washed in for 10 min to block HCN channels. ZD7288 was applied at a 10 μM concentration in a set of control experiments in order to rule out dose-dependent, off-target effects. In another set of control experiments, 20 μM ZD7288 was added to the normal K-gluconate internal solution for cell-specific, post-synaptic HCN channel inhibition. Tetrodotoxin-citrate (1 μM; Sigma, St. Louis, MO, USA) was used to block AP mediated synaptic transmission for the analysis of mEPSCs. All drugs were bath applied unless otherwise stated, with each neuron serving as its own control.

### Statistics

Statistical analysis of electrophysiological data was performed using GraphPad Prism 6 (La Jolla, CA, USA). Data are expressed as either mean ± SEM or dots representing each individual data point. Traces shown are the average of 10 sweeps. Sample size *(n)* is the number of cells used for each experiment, with a minimum of three animals used per group. Statistical comparisons of responses before and during drug application was performed using a one- or two-tailed Student’s *t*-test, or Two-way ANOVA with a Sidak correction for multiple comparisons. For all tests, *p* < 0.05 was considered significant.

Unbiased hierarchical cluster analysis using the Ward method with a Euclidean distance metric was performed with R-based software (Wessa, 2012), and partial least squared enhanced discrimination analysis (PLS-EDA) was performed using the Excel add-in Multibase package (Numerical Dynamics, Japan). The cutoff for determining the number of clusters was determined by the exponential decay in the Euclidean distance between branch points. All variables were normalized to their *z* score for PLS-EDA. Aside from frequency adaptation and firing frequency (*r*^2^ = 0.42), all properties used for cluster analysis were weakly correlated to one another (*r*^2^ < 0.23).

## Results

### Unbiased Clustering of Mature L1 INs

**Figure 1:**
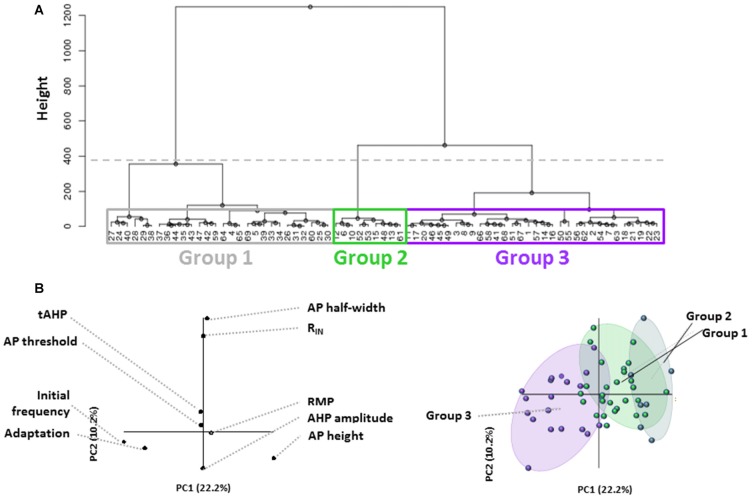
Unbiased identification of physiological distinct layer 1 (L1) interneuron (IN) populations. **(A)** Dendrogram generated from hierarchical cluster analysis of electrophysiological properties of L1 cells. The dashed horizontal line indicates the cutoff for heterogeneous groups based on the exponential decay in the Euclidean distance between consecutive branch points. Boxes indicate the three groups identified. Colors correspond to Panel **(B)** right. Each terminal branch represents a recorded L1 IN, each indicated by a number. **(B)** Partial least squares enhanced discrimination analysis (PLS-EDA) was used to ascertain the defining properties for identified groups. The contribution of assessed properties to each principal component (PC) is illustrated in the graph to the left. Correlation of individual cells plotted on the same axes (right graph) reveals that initial firing frequency and frequency adaptation most strongly identify Group 3, with Groups 1 and 2 being distributed along the same PC axis.

In this study, we analyzed electrophysiological properties of L1 INs in rat AGm in order to identify and characterize distinct cell groups within L1. Using hierarchical clustering based on a set of eight active and passive membrane properties which are commonly used to differentiate neuronal subtypes, we were able to identify three distinct L1 cell types in the adult AGm (Figure 1A). Each terminal dendrogram branch represents an individual L1 neuron. The height (Figure 1A, Y-axis) of branching points reflects the physiological similarity of groups/cells with larger height values indicating a larger degree of dissimilarity. PLS regression was then performed to confirm that the clusters represented physiologically distinct groups and to identify the key differentiating characteristics. The contribution of assessed properties to each PC is shown in Figure 1B, left. When the correlation of individual cells was plotted on the same axes (Figure 1B, right) initial firing frequency and frequency adaptation most strongly identify Group 3, with Groups 1 and 2 being distributed along the same PC axis. PLS analysis suggested cell clusters were most strongly differentiated based on initial firing frequency and spike frequency adaptation.

**Figure 2:**
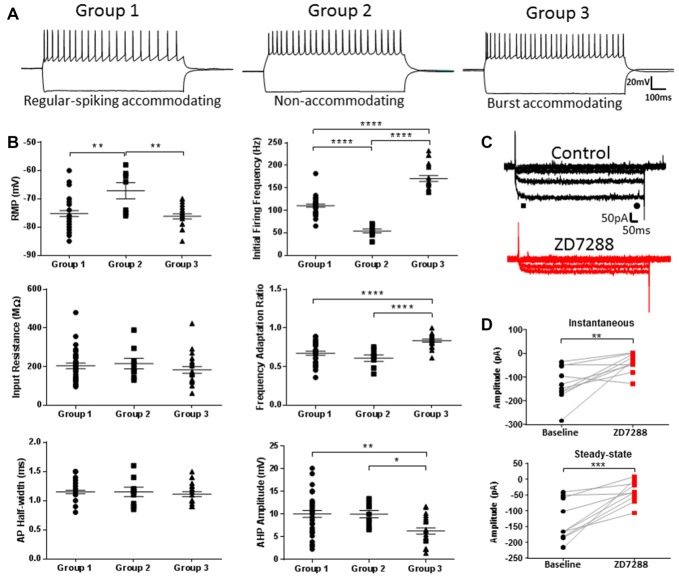
Characterization of identified L1 IN populations. **(A)** Representative examples of the firing patterns observed for each of the cell groups shown in Figure 1. **(B)** Quantitative comparison of intrinsic properties in the cell groups including initial firing frequency and frequency adaptation, consistent with PLS-EDA results. **(C)** Example traces of hyperpolarization-activated current (*I*_h_) recordings before (black, top) and after (red, bottom) wash-on of 20 μM ZD7288. Instantaneous and steady-state currents were measured at the peak after current onset and immediately prior to current offset, as indicated by the black bar and circle, respectively. **(D)** Plots showing peak instaneous and steady state current amplitudes before and after ZD7288. Hyperpolarization-activated, cyclic nucleotide-gated, non-specific cation (HCN) channel inhibition produced a decrease in both currents indicative of the presence of a small amplitude *I*_h_ current in L1 INs. *p < 0.05, **p < 0.01, ***p < 0.001, ****p < 0.0001, Tukey’s post-test. Each shape represents an individual cell. Error bars are mean ± SEM.

Quantitative comparison of firing properties between groups supported the finding that firing frequency and adaptation defined each cell type (Figure 2A; Initial firing frequency—Group 1: 110.4 ± 3.5 Hz, Group 2: 54.2 ± 4.3 Hz, Group 3: 170.7 ± 6.4 Hz; *p* < 0.0001, 1-way ANOVA; Spike frequency adaptation—Group 1: 0.67 ± 0.03, Group 2: 0.61 ± 0.04, Group 3: 0.84 ± 0.02; *p* < 0.0001, 1-way ANOVA). No differences in AP threshold, AP height or tAHP were seen between groups. Based on their intrinsic properties, the three groups were classified as Group 1, regular spiking (RS), Group 2, non-accommodating (NA) and Group 3, burst accommodating (BA), respectively (Figure 2B).

Figure 2C shows responses of a L1 IN to a family of hyperpolarizing voltage steps under control condition and after HCN channel blockade with ZD7288. Instantaneous and steady-state currents associated with *I*_h_ are shown in Figure 2C. Using the HCN channel inhibitor ZD7288, we were able to confirm the presence of small amplitude *I*_h_ in L1 INs (Figure 2D; Instantaneous—Control: 128.57 ± 23.71 pA, ZD7288: 39.15 ± 12.86 pA; *p* < 0.01, paired *t*-test; Steady-state—Control: 132.97 ± 20.19 pA, ZD7288: 38.73 ± 10.92 pA; *p* = 0.0001, paired *t*-test). The measured *I*_h_ amplitude was small compared to currents previously observed in L5 pyramidal neurons (PNs; Albertson et al., 2011) and GABAergic INs (Albertson et al., 2017). The voltage “sag” response characteristic of *I*_h_ was also quantified to assess differences in functional HCN channel expression between groups. A small HCN channel mediated sag was observed in each cell-type, however no differences were seen between groups (data not shown; Time-current amplitude interaction: *p* = 0.9822, 2-way ANOVA).

### Differential Modulation of the Excitability of Mature L1 INs by HCN Channels

**Figure 3:**
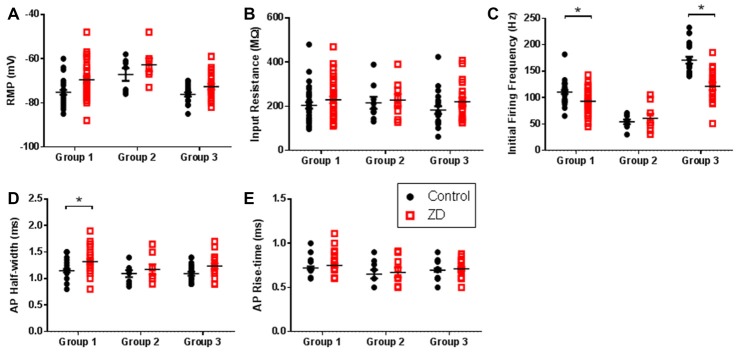
HCN channel inhibition exerts cell-type specific effects on intrinsic excitability. Scatter plots showing **(A)** the resting membrane potential (RMP), **(B)** input resistance (*R*_IN_), **(C)** initial firing frequency, **(D)** action potential (AP) half-width and **(E)** AP rise time of cells in each group before (black circles) and after (red squares) wash-on of the HCN channel antagonist ZD7288. Group 1 showed the largest effects whereas Group 2 cells displayed no modulation of intrinsic excitability by HCN channels. *p < 0.05, paired *t*-test. Each shape represents an individual cell. Error bars are mean ± SEM.

HCN channels have been shown to modulate various intrinsic neuronal properties in a cell-specific manner (Robinson and Siegelbaum, 2003; Biel et al., 2009). Having established the presence of *I*_h_, we bath applied ZD7288 to determine the role of HCN channels in modulating the intrinsic excitability of identified AGm L1 IN groups. As shown in Figures 3A,B, application of ZD7288 did not produce a significant RMP hyperpolarization (RS—Control: −75.2 ± 1.0 mV, ZD7288: −69.5 ± 1.5 mV; *p* > 0.05, 1-tailed *t*-test; NA—Control: −67.1 ± 2.9 mV, ZD7288: −62.7 ± 2.9 mV; *p* > 0.05, 1-tailed *t*-test; BA—Control: −76.2 ± 0.9 mV, ZD7288: −72.6 ± 1.4 mV; *p* > 0.05, 1-tailed *t*-test) or increase in *R*_IN_ (RS—Control: 204.26 ± 14.7 MΩ, ZD7288: 229.23 ± 15.6 MΩ; *p* > 0.05, 1-tailed *t*-test; NA—Control: 215.46 ± 27.2 MΩ, ZD7288: 227.57 + 27.5 MΩ; *p* > 0.05, 1-tailed *t*-test ; BA—Control: 182.86 ± 17.2 MΩ, ZD7288: 220.4 ± 17.6 MΩ; *p* > 0.05, 1-tailed *t*-test) for any cell type. The initial firing frequency of both RS and BA groups were decreased following ZD7288 application (RS—Control: 110.4 ± 3.5 Hz, ZD7288: 92.9 ± 4.2 Hz; *p* < 0.01, Holm-Sidak test; BA—Control: 170.7 ± 6.4 Hz, ZD7288: 121.4 ± 7.1 Hz; *p* < 0.0001, Holm-Sidak test), with no effect observed on NA neuron firing rate (Control: 54.2 ± 4.3 Hz, ZD7288: 60.8 ± 8.5 Hz; *p* = 0.5, Holm-Sidak test; Figure 3C). In addition, HCN channel inhibition caused a significant increase in the AP half-width of RS neurons (Control: 1.15 ± 0.03 ms, ZD7288: 1.32 ± 0.1 ms; *p* < 0.01; Holm-Sidak test) without affecting the AP rise time (Control: 0.72 ± 0.02 ms, ZD7288: 0.75 ± 0.02 ms; *p* = 0.32, Holm-Sidak test; Figures 3D,E).

**Figure 4:**
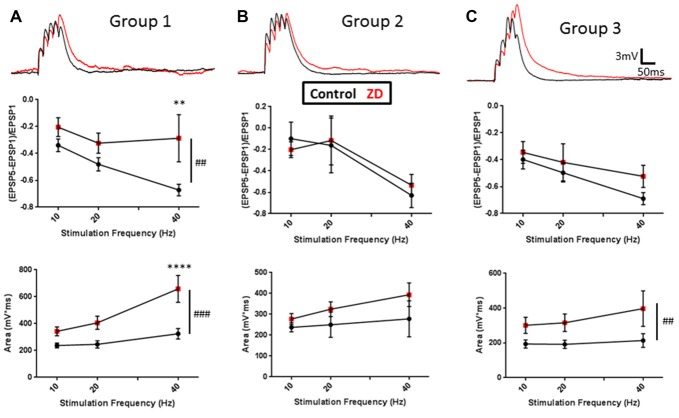
Cell-group dependent effects of HCN channel inhibition on synaptic excitability. **(A)** Upper: representative examples of evoked EPSPs before (black) and after (red) bath application of ZD7288. Middle: plots of EPSP ratios before (black) and after (red) ZD7288 wash-on. Lower: plot of total area under the curve (AUC) of EPSPs evoked under control conditions (black) and in the presence (red) of ZD7288. **(B,C)** Same as in **(A)** but for Groups 2 and 3, respectively. As with intrinsic excitability, group-specific effects were observed, with Group 1 showing the largest effects and Group 2 cells displaying no modulation by HCN channels. ^##^*p* < 0.01, ^###^*p* < 0.001, 2-way ANOVA. **p < 0.01, ****p < 0.0001, Dunn-Sidak’s post-test. Error bars are mean ± SEM.

Extensive work has also described a role for HCN channels in modulating the integration of synaptic inputs to neurons (Stuart and Spruston, 1998; Williams and Stuart, 2000; Berger et al., 2001; Albertson et al., 2011). These studies have shown that the density and distribution of HCN channels across a cell’s processes can affect frequency dependent filtering and spatial integration of synaptic inputs. To characterize the contribution of HCN channels to the synaptic excitability of L1 INs, we evaluated the effect of ZD7288 application on the integration of synaptic responses evoked at 10, 20 and 40 Hz (Figure 4). Synaptic integration was evaluated by quantifying EPSP amplitude summation as well as the total area under the curve of evoked activity (AUC). Specimen records of EPSPs evoked in RS, NA and BS cells are shown in upper portions of Figures 4A–C, respectively. In RS Group 1 cells, inhibition of HCN channels significantly increased synaptic summation (*p* < 0.01, 2-way ANOVA) and AUC (*p* < 0.0001, 2-way ANOVA), particularly with 40 Hz input (Summation: *p* < 0.01, Sidak post-test; AUC: *p* < 0.0001, Sidak post-test; Figure 4A, middle and lower). In contrast, HCN channels inhibition did not have any effect on the summation (*p* = 0.9277, 2-way ANOVA) or AUC (*p* = 0.0854, 2-way ANOVA) of EPSPs in Group 2 NA cells (Figure 4B). The synaptic integration of BS Group 3 cells demonstrated moderate HCN channel modulation, showing a significant increase in the AUC (*p* < 0.01, 2-way ANOVA), but no observable increase in the summation of EPSPs (*p* = 0.1729, 2-way ANOVA) following ZD7288 application (Figure 4C). In combination with the distinct effects of ZD7288 on the intrinsic properties of identified groups, these results suggest HCN channels modulate the excitability of RS and BA cells, but do not significantly affect NA neurons in the adult AGm.

In addition to effects on neuronal excitability, HCN channels have also been shown to modulate presynaptic neurotransmitter release in certain cells populations (Santoro et al., 1997; Southan et al., 2000; Cuttle et al., 2001; Aponte et al., 2006). To determine if effects observed upon ZD7288 application were due to a postsynaptic effect, we examined presynaptic neurotransmitter release by assessing the amplitude and frequency of miniature EPSCs onto L1 INs, as well as the paired-pulse ratio (PPR) of evoked EPSCs. No changes were observed in the frequency (Control: 0.92 ± 0.18 Hz, ZD7288: 0.98 ± 0.18 Hz; *p* > 0.999, Kolmogorov-Smirnov test) or amplitude (Control: 20.11 ± 0.99 pA, ZD7288: 10.09 ± 0.99 pA; *p* = 0.998, Kolmogorov-Smirnov test) of miniature EPSCs (data not shown). Moreover, ZD7288 had no effect on the PPR of EPSCs evoked at 25 ms (Control: 0.83 ± 0.1, ZD7288: 0.95 ± 0.1; *p* = 0.143, paired *t*-test), 50 ms (Control: 0.87 ± 0.1, ZD7288: 0.96 ± 0.1; *p* = 0.237, paired *t*-test) or 100 ms (Control: 0.85 ± 0.1, ZD7288: 0.97 ± 0.1; *p* = 0.152, paired *t*-test) intervals, suggesting any effects of ZD7288 were post synaptic. To further confirm a post-synaptic mechanism of action for ZD7288, summation experiments were performed using a modified intracellular solution containing either Cs^+^, a non-specific HCN channel blocker, or ZD7288 to specifically block post-synaptic HCN channels. Under both conditions, the effects of ZD7288 on 40 Hz EPSP summation (Cs^+^—Control: −0.39 ± 0.4, ZD7288: −0.75 ± 0.1; *p* = 0.30, paired *t-test;* Intracellular ZD7288—Control: −0.61 ± 0.1, ZD7288: −0.49 ± 0.1; *p* = 0.08, paired *t*-test) were abrogated (data not shown), suggesting a post-synaptic basis for the changes in synaptic excitability observed following HCN channel inhibition.

### Classification of L1 INs during Development

It is well known that synaptic activity during development shapes cortical connectivity effecting changes that will persist into adulthood. Recent work has shown that HCN channel expression and function can display developmental changes, conferring varying patterns of excitability modulation (Surges et al., 2006; Bender and Baram, 2008; Cho et al., 2011; Seo et al., 2015). We therefore sought to determine if the effects of HCN channels on L1 IN excitability were developmentally regulated.

CR cells constitute the majority of the neuronal population found in L1 during the first postnatal week (Bradford et al., 1978; Chun and Shatz, 1989; Zhou and Hablitz, 1996b). Since the presence of CR cells is transient, virtually disappearing by postnatal day 14 (P14), the developmental time course of HCN channel effects was characterized from P10 to P21 in order to assess cell-types in which the effect of HCN channels could be evaluated into adulthood. Under these restrictions we could reasonably presume that neurons recorded at the youngest age would ultimately develop into one of the cell-types identified in adult animals. Based on the time course of changes in the intrinsic properties of L1 INs (data not shown), developmental data was grouped into three-day bins for analysis: P10–12, P13–15, P16–18 and P19–21.

**Figure 5:**
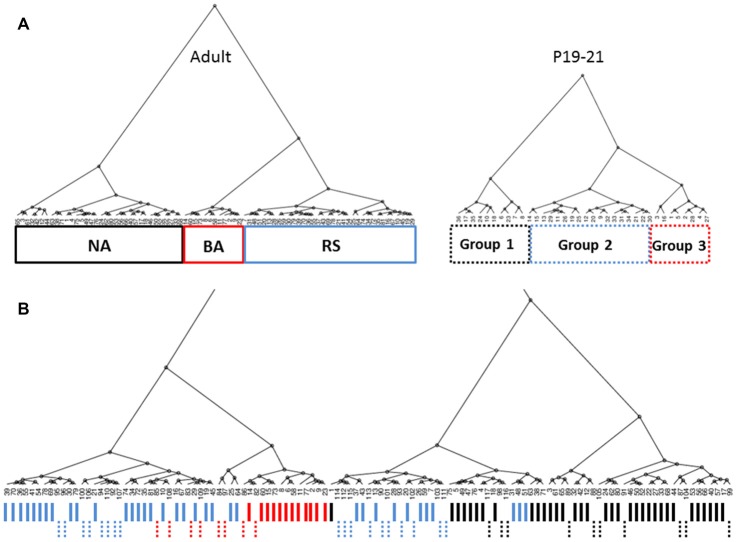
Cell groups can be tracked throughout development. **(A)** Dendrograms generated by unbiased hierarchical cluster analysis of the electrophysiological properties of cells recorded from adult animals (left) and P19–21 (right) animals. Three distinct groups are identified in both age groups. As with the adult groups, PLS analysis identified initial firing frequency and frequency adaptation as key determinants of neuronal grouping at P19–21. **(B)** Combined analysis of Adult and P19–21 animals reveals overlap between cell groups identified at chronologically sequential ages, indicating cell groups in adult animals can also be identified at younger ages. Solid and dashed lines indicate cells from adult and P19–21 animals, respectively. Line colors in **(B)** coincide with the line color of group outlines shown in **(A)**.

**Figure 6:**
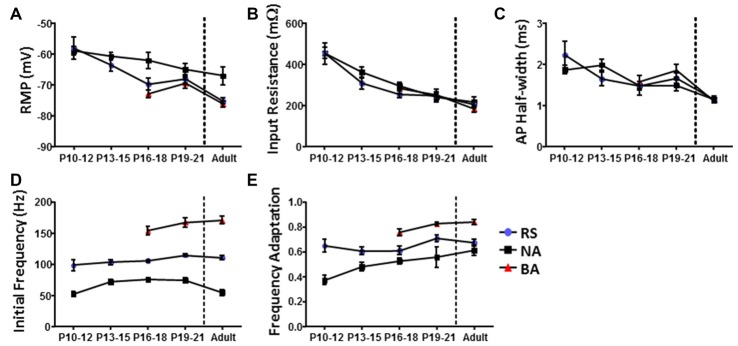
Identified L1 IN groups display distinct intrinsic properties throughout development. Graphs illustrating differences in the **(A)** RMP, **(B)** *R*_IN_, **(C)** AP half-width, **(D)** initial firing frequency and **(E)** frequency adaptation of cells in each group as a function of development. Comparison of intrinsic properties across groups suggests a specific distinction between non-accommodating (NA) cells and the other groups. Error bars are mean ± SEM.

Currently available methods do not allow for chronic whole-cell recordings from a single neuron across the developmental range investigated here. Therefore, identification of specific cell-types throughout development was performed using cluster analysis and PLS as was done for cells from adult animals. Cluster analysis was first performed within each 3 day group to definitively identify cell clusters at each age. Figure 5A shows dendrograms generated by unbiased hierarchical cluster analysis of the electrophysiological properties of cells recorded from adult animals (left) and P19–21 (right) animals. To confirm identified groups represented the same cell populations throughout development, cluster analysis was then performed in reverse chronological order combining sequential age groups, as shown in Figure 5B for the Adult and P19–21 groups. Using this method, the RS and NA clusters identified in adult animals could be traced throughout development back to P10, with BA cells emerging at P16. As with the adult groups, PLS analysis identified initial firing frequency and frequency adaptation as key determinants of neuronal grouping at all ages, consistent with identified clusters representing cell types which are present throughout development. Comparison of intrinsic properties across groups revealed a specific distinction between NA cells and the other groups (Figure 6). Specifically, NA cells displayed a more depolarized RMP (Figure 6A) and lower firing frequency (Figure 6D) throughout development. BA cells exhibited a significantly higher firing frequency than both RS and NA cells (Figure 6D), but did not differ from RS cells in any other properties. Due to the similarity of their intrinsic properties, it is possible that BA neurons represent a subset of RS cells which developmentally differentiates based on the expression of channels which facilitate the higher firing frequency.

### Distinct Patterns of HCN Channel Excitability Modulation in L1 Throughout Development

**Figure 7:**
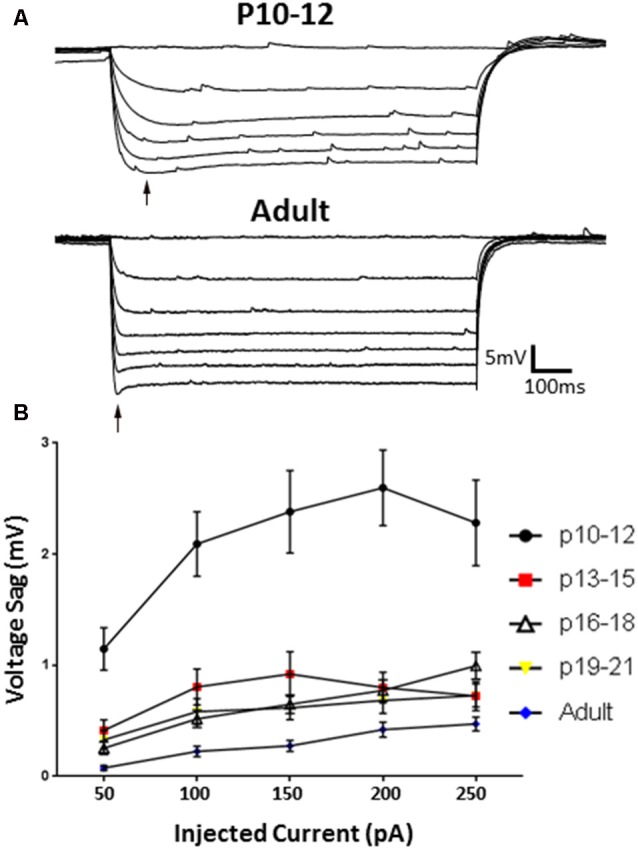
HCN channel-mediated sag responses change with development. **(A)** Example traces of the sag responses from a P10–12 IN (top) and adult (bottom) animals. **(B)** The *I*_h_-associated voltage sag is significantly larger at P10–12, and significantly smaller in adult cells, indicating developmental regulation of HCN-mediated effects. Black arrow indicates sag. Error bars are mean ± SEM.

Having confirmed identified clusters persist during development, we next assessed whether L1 INs displayed developmental changes in HCN channel mediated sag amplitude (Figure 7). Specimen records of responses to a series of hyperpolarizing current pulses are shown in Figure 7A for a P10–12 (upper) and adult (lower) group neuron. INs recorded at P10–12 displayed a significantly larger voltage sag then observed at all other ages (*p* < 0.0001, Tukey’s test), with adult neurons displaying the smallest sag (Figure 7B; *p* < 0.01, Tukey’s test). Sag amplitude stabilized following P12, with no difference being observed between any other developmental age groups. At P13–15 the voltage sag of NA neurons was significantly larger than that of RS neurons (*p* < 0.0001, 2-way ANOVA), though no difference between groups persisted throughout the entire developmental time course.

**Figure 8:**
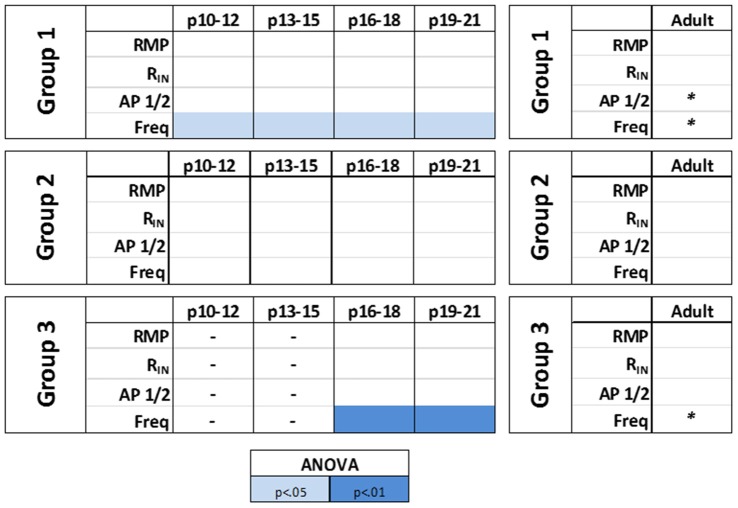
HCN channels differentially modulate the intrinsic excitability of identified L1 IN groups over development. A summary of the effects of HCN channels blockade on the intrinsic properties of identified cell groups throughout development. A small contribution of HCN channels to the intrinsic excitability of L1 INs was observed with HCN channel inhibition producing a significant decrease in the initial frequency of regular spiking (RS) and burst accommodating (BA) neurons across their developmental ranges but not affecting the excitability of NA cells at any age. Statistical comparison was performed on values obtained before and after ZD7288 wash-on. *p < 0.05, paired *t*-test.

The effects of HCN channels on intrinsic excitability throughout development were then examined. As summarized in Figure 8, HCN channel inhibition resulted in a significant decrease in the initial frequency of RS and BA neurons across their developmental ranges, but did not affect the firing of NA cells. Consistent with data from adult cells, application of ZD7288 did not produce membrane hyperpolarization or an increase in *R*_IN_ in any group during development.

**Figure 9:**
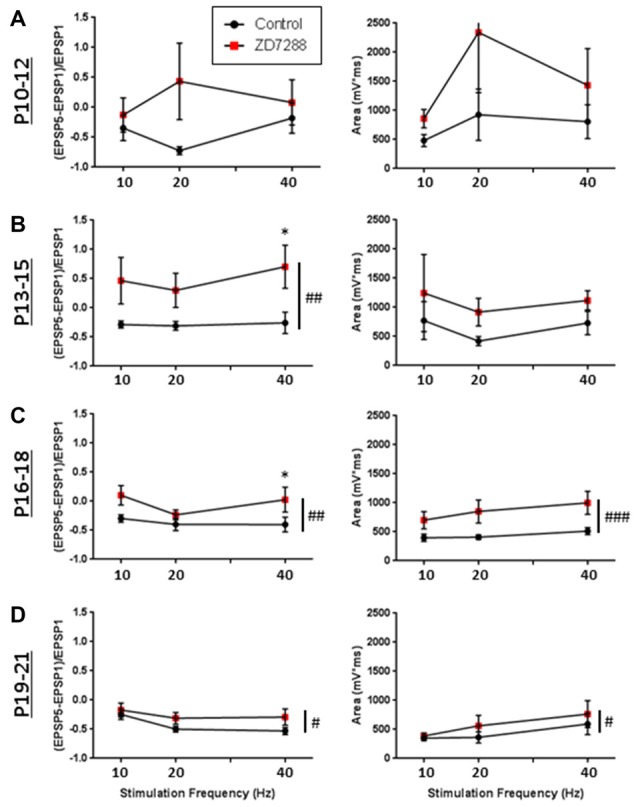
Developmental modulation of synaptic integration by HCN channels. **(A)** Graphs quantifying the effects of HCN channel blockade (red squares) on EPSP summation (left) and the total AUC of evoked EPSPs (right) in RS Group 1 cells recorded at p10–12. **(B–D)** Same as in **(A)**, but for cells recorded at p13–15 **(B)**, p16–18 **(C)**, and p19–21 **(D)**, showing that in RS cells, modulation by HCN channels increases developmentally. ^#^*p* < 0.05, ^##^*p* < 0.01, ^###^*p* < 0.001, 2-way ANOVA. *p < 0.05, Dunn-Sidak’s post-test. Error bars are mean ± SEM.

**Figure 10:**
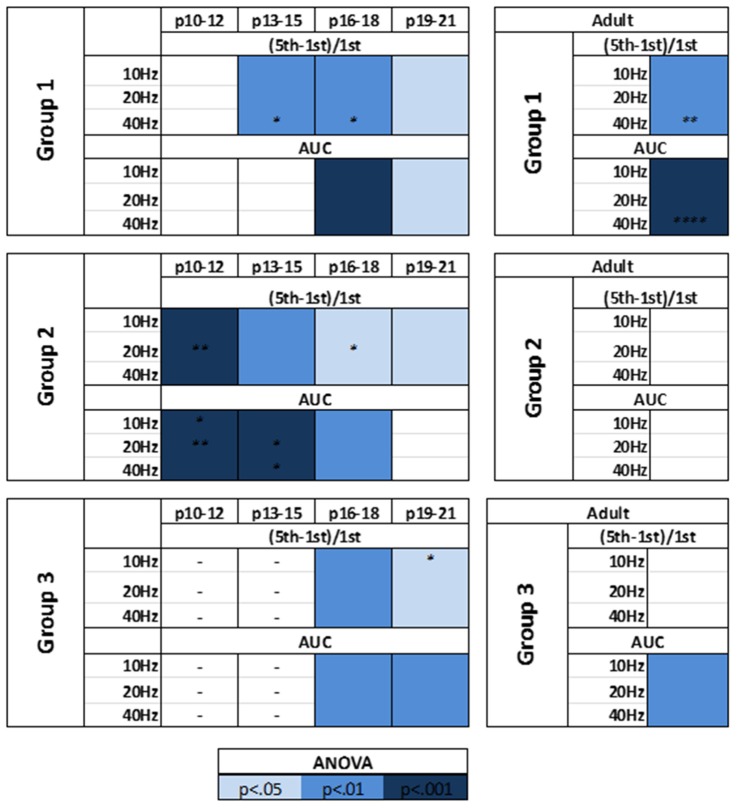
HCN channels display distinct patterns of synaptic excitability modulation in identified L1 IN groups throughout development. A summary of the effects of HCN channels blockade on the synaptic excitability of identified cell groups throughout development. Viewing developmental data together with data generated from adult cells, clear developmental patterns of HCN channel modulation emerge, with modulation increasing and decreasing with age in RS and NA cells, respectively. Statistical comparison was performed on values obtained before and after ZD7288 wash-on. *p < 0.05, **p < 0.01, ****p < 0.0001, Dunn-Sidak’s post-test.

The modulatory effects of HCN channels on synaptic integration throughout development were also examined. In RS cells, modulation by HCN channels increased developmentally (Figure 9). HCN channel inhibition had no effect on synaptic excitability at the youngest age recorded (Figure 9A), caused a significant increase in summation but not AUC at P13–15 (Figure 9B), and from P16 to P21 significantly increased both summation and AUC (Figures 9C,D). In stark contrast, we found the effects of HCN channel inhibition displayed a pattern of decreasing significance throughout development in NA neurons (Figure 10). In NA cells at P10–12 inhibition of HCN channels caused the largest change in summation and AUC seen for any group at any age. Summation and AUC were both significantly affected by HCN channel inhibition for NA cells at P13–15 and P16–18, with the magnitude of observed changes becoming progressively smaller with age. In P19–21 NA cells, inhibition of HCN channels caused a small, significant increase in summation, but had no effect on AUC. BS cells demonstrated modulation by HCN channels similar to that seen in RS cells. Both the summation and AUC of EPSPs were significantly increased in BS neurons at P16–18 and P19–21 (Figure 10). Viewing developmental data together with data generated from adult cells, clear developmental patterns of HCN channel modulation emerge, with modulation increasing and decreasing with age in RS and NA cells, respectively (Figure 10). As with cells from mature animals, HCN channel inhibition had no effect on the frequency of mEPSCs, again suggesting changes seen in the presence of ZD7288 are due to a postsynaptic effect.

### Morphology of L1 IN Groups Is Correlated with Physiology

**Figure 11:**
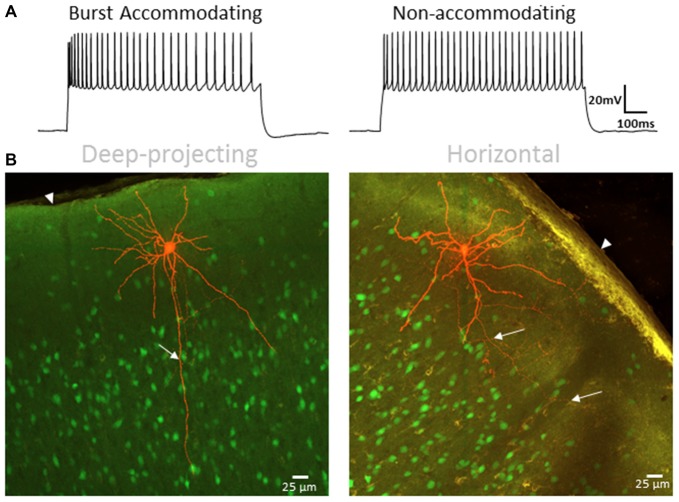
L1 IN physiology correlates with morphology. **(A)** Specimen records showing BA (left) and NA (right) firing patterns used for correlation with axonal projection type. **(B)** Representative images of a deep-projecting (left) and horizontally-confined (right) L1 INs. Labeled cells are shown in red with other VGAT-positive cells shown in green. White arrows indicate axonal projections whereas arrowheads indicate the pial surface. A significant correlation between type of axon projection and physiology was identified (*χ*^2^: *p* < 0.01), with a distinct deep projecting axon being identified in a larger percentage of BA cells (BA = 11 of 14, NA = 1 of 8), while a greater portion of NA cells were identified as horizontally projecting (BA = 1 of 14, NA = 3 of 8). Scale bar = 25 μm.

Morphological analysis was performed in a subset of BA and NA cells (Figure 11A) from adult animals to further validate that the identified cell clusters represent distinct neuronal groups. Blinded to physiological grouping, *post hoc* reconstructed L1 INs were morphologically classified as either deep projecting or horizontally projecting, as described by Jiang et al. (2013) and Lee et al. (2015) (Figure 11B). A significant correlation between morphology and physiology was identified (*χ*^2^: *p* < 0.01), with a distinct deep projecting axon being identified in a larger percentage of BA cells (BA = 11 of 14, NA = 1 of 8), while a greater portion of NA cells were definitively identified as horizontally projecting (BA = 1 of 14, NA = 3 of 8). Cells for which a clear classification could not be determined were not included in either group. This finding is consistent with previous data suggesting the physiological properties of L1 INs can be used to identify discrete cell groups (Wozny and Williams, 2011; Jiang et al., 2013).

## Discussion

In the present study we investigated the modulatory influence of HCN channels on the intrinsic and synaptic excitability of AGm L1 INs throughout development. Our studies revealed two distinct groups which display divergent patterns of HCN channel modulation during development. We found that HCN channel inhibition altered the repetitive firing properties of RS neurons across every age, whereas no effects on the intrinsic excitability of NA neurons were seen. RS cells also displayed a pattern of gradually increasing modulation of synaptic excitability with age. Conversely, NA neurons evidenced decreased modulation by HCN channels over time. A third cell-type physiologically characterized as BS neurons was also identified beginning at p16. BS cells displayed morphological and physiological characteristics similar to those of RS cells. The influence of HCN channel inhibition on the excitability of BS cells closely resembled that of RS cells, suggesting BS cells may represent a subset of RS neurons differentiated by channel expression.

### Effect of IN Excitability on Cortical Output

In the neocortex, GABAergic INs are the primary source of inhibition and serve to regulate glutamate-driven network output (Markram et al., 2004; Silberberg, 2008; Hu et al., 2014). Extensive studies characterizing cortical GABAergic INs have revealed multiple IN classes which display unique intrinsic properties and synaptic targets (DeFelipe, 1993; Cauli et al., 1997; Kawaguchi and Kubota, 1997; Gupta et al., 2000). Due to these functional differences, INs display class-specific roles in regulating network excitability via mechanisms such as spike timing control and modulation of synaptic integration (Pinto et al., 2000; Pouille and Scanziani, 2001; Wehr and Zador, 2003; Gabernet et al., 2005). Cortical output via PNs is strongly directed by the integration of synaptic inputs to PN dendrites. The ability of synaptic inputs to affect PN firing requires spatiotemporal integration of multiple signals in order to facilitate EPSP summation and propagation to the soma (Magee, 2000). This synaptic summation involves the additive effect of synchronous EPSPs, as well as the divisive integration of inhibitory, GABAergic input (Palmer et al., 2012b; Chiu et al., 2013; Lee et al., 2014). As such, the relative timing and kinetics of inhibitory and excitatory inputs directly affect PC firing and synaptic plasticity (Golding et al., 2002; Larkum et al., 2009).

HCN channels have been shown to modulate the excitability of CR cells (Kilb and Luhmann, 2000) and affect the intrinsic and synaptic excitability of diverse cortical INs (Albertson et al., 2011, 2017). HCN channels can be active at rest, contributing to the RMP and conductance of the membrane in a cell-type specific manner (Pape, 1996; Lupica et al., 2001; Nolan et al., 2003; Day et al., 2005; Meuth et al., 2006). Although not a canonically associated function, HCN channels can also modulate repetitive firing properties via attenuation of AP half-width and mAHP duration (Tanaka et al., 2003; Kouranova et al., 2008; Cho et al., 2011). Of note, current methods have not been able to elucidate the subcellular localization of HCN channels which strongly determines their contribution to neuronal activity and excitability. As with PNs, spatiotemporal integration of multiple signals is necessary for synaptic inputs to modify the firing patterns of INs. Many studies have characterized a role for HCN channels in restricting the spatiotemporal integration of synaptic inputs (Stuart and Spruston, 1998; Williams and Stuart, 2000; Berger et al., 2001; Albertson et al., 2011). Specifically, HCN channels have been shown to attenuate the amplitude and duration of EPSPs, and act as a coincidence detector by preferentially facilitating the summation of spatially distributed inputs which arrive within a narrow temporal window (Dembrow et al., 2015). HCN channels can therefore act as key modulators of neuronal firing in response to synaptic input through effects on both intrinsic and synaptic neuronal excitability. HCN modulatory effects in L1 INs could subsequently influence the integration of synaptic inputs on PC dendrites, potentially altering cortical output.

In addition to the sparse IN population, L1 also contains dense axonal and dendritic plexuses. PNs in cortical layers 2/3, 5 and 6 are the sole output neurons of the cortex, and are characterized by their long, apical dendrites which ramify extensively in superficial layers, particularly L1 (Gottlieb and Keller, 1997; Larsen and Callaway, 2006; Oberlaender et al., 2011). Inputs from multiple pathways converge onto the dendritic trees of PNs in L1 (Vogt, 1991; Mitchell and Cauller, 2001; Llinás et al., 2002; Arroyo et al., 2012; Cruikshank et al., 2012). L1 INs are also targeted by these inputs and are uniquely positioned to directly inhibit the apical tufts of pyramidal cells (Hestrin and Armstrong, 1996; Zhou and Hablitz, 1996a; Larkum and Zhu, 2002; Zhu and Zhu, 2004), which are critical for the spatiotemporal integration of their multiple inputs (Vogt, 1991; Williams, 2004; Larkum et al., 2009; Palmer et al., 2012a). As such, the intrinsic excitability and synaptic integration properties of L1 cell populations are pivotal in maintaining the network dynamics underlying cortical information processing.

Given that inputs to L1 potentially form synapses on both PN dendrites and L1 INs, PN dendrites can receive a combination of excitatory input and feedforward inhibition from activation of a single afferent fiber (Mitchell and Cauller, 2001; Llinás et al., 2002; Larkum et al., 2009). Following classical Hebbian plasticity, synaptic connectivity is strongly modified by changes in the input-output dynamics of synaptically coupled cells. Early in development, the correlation between a synaptic input and the firing of the post-synaptic cell will influence the strengthening or pruning of that synapse (Purves and Lichtman, 1980; Shatz, 1990; Katz and Shatz, 1996). The excitability of L1 INs is central to determination of the timing and kinetics of their synaptic output, which modulates the integration of spatiotemporally distinct inputs on PN dendrites. In this way, the excitability of L1 INs during development can affect the formation of cortical connectivity patterns which produce information circuits that will persist into maturity.

### Different Contributions of IN Subtypes to Cortical Network Activity

The L1 IN subtypes we have identified coincide well with other reported classification dichotomies: deep projecting, accommodating cells have been identified as elongated neurogliaform cells (ENGCs) and horizontal, NA cells as single-bouquet cells (SBCs; Kubota et al., 2011; Jiang et al., 2013). These studies have characterized these L1 IN subtypes, identifying distinct patterns of synaptic connectivity. Of particular relevance to our findings, opposing effects on PC firing have been reported for these identified cell types (Larkum, 2013). Specifically, Jiang et al. (2013) found that activation of SBCs resulted in an increase in PC firing through disinhibition of L2/3 INs, whereas NGC activation produced a significant decrease in PC firing via synchronization of L2/3 IN firing which resulted in a decrease of dendritic spikes.

Our recent work has shown that in addition to regulating the intrinsic excitability of PCs and excitatory network activity, HCN channels also constrain GABAergic network activity (Williams and Hablitz, 2015). The conflicting effect of SBCs and NGCs on PC firing could be of particular importance when establishing synaptic connectivity patterns during cortical development. The differential patterns of HCN channel effects could facilitate complementary roles for the two IN classes. HCN channel-mediated attenuation of NGC/NA cells’ synaptic excitability early in development would restrict temporal EPSP summation in those cells. In theory, this effect would attenuate the summation of temporally separate inputs and decrease the probability of AP elicitation, thereby preventing the correlation of distinct afferents and possibly prompting the pruning of those synapses. Conversely, spatially disparate, yet synchronous inputs to NA neurons would have an increased probability of eliciting an AP under these conditions, subsequently producing an increase in PC firing through inhibition of L2/3 INs. By providing synchronous input to NA cells, distinct afferents could become correlated through synaptic strengthening as a result of direct correlation to the firing of both NA cells and PCs. Through this mechanism, synaptic connectivity and correlation patterns could be established which would be stabilized by the formation of perineuronal nets throughout development. With maturity and stabilization of synaptic connectivity and synchronicity, the specificity of synaptic integration conferred by HCN channels would be of less importance than during development. Furthermore, the lack of HCN channel-mediated EPSP attenuation in adults would increase the probability of synaptic input triggering an AP.

In RS cells, the lack of HCN channel modulation early in development would enhance the probability of AP firing in response to synaptic input. Under these conditions, synaptic input causing activation of a RS cell would produce a decrease in PC firing through synchronization of L2/3 mediated inhibition. With increased HCN channel modulation in adults, the summation of synaptic inputs onto RS cells would be attenuated, thereby decreasing the probability of eliciting an AP in a RS cell and consequently decreasing inhibition of PC firing. In theory, the inverse developmental patterns of HCN channel effects in NA and RS cells convey synaptic dynamics which are complementary in terms of network dynamics. As a consequence of HCN channel function early in development, temporally disparate synaptic inputs to L1 INs are likely to exert only a small influence on PC firing, preferentially causing a decrease in firing rate. Synchronous input to L1 during this time, however, would likely cause a significant increase in PC firing due to the modulatory effect of HCN channels on NA cells’ synaptic integration. Theoretically, the opposite would then hold true under the conditions of altered HCN channel modulatory effects in adults. It is likely that asynchronous input to L1 in the mature cortex would still induce only a modest change in PC firing; however the effect would more likely be an increase in PC firing. Conversely, due to the effects of HCN channels on RS cells’ synaptic integration, synchronous input to L1 would more likely increase PC firing early in development. In this way, HCN channels could potentially serve to refine synaptic connectivity and dynamics during development and constrain the excitability of the mature cortex. Further experiments are necessary to directly test this paradigm, as this study has not investigated how the modulatory effects of HCN channels on L1 INs affects PC firing or di-synaptic inputs to PCs. Given that electrical coupling has been observed between L1 INs (Muralidhar et al., 2014; Yao et al., 2016), further studies are also necessary to determine if intergroup coupling exists or if coupling is exclusively between physiologically similar cells, creating isolated synchronized networks.

## Author Contributions

ASB and JJH designed the research and wrote the manuscript. ASB conducted experiments and analyzed the data.

## Conflict of Interest Statement

The authors declare that the research was conducted in the absence of any commercial or financial relationships that could be construed as a potential conflict of interest.
